# Insights Into Excess Mortality During the First Months of the COVID-19 Pandemic From a Rural, Demographic Surveillance Site in Bangladesh

**DOI:** 10.3389/fpubh.2021.622379

**Published:** 2021-07-29

**Authors:** Syed Manzoor Ahmed Hanifi, Sayed Saidul Alam, Sanjida Siddiqua Shuma, Daniel D. Reidpath

**Affiliations:** Health Systems and Population Studies Division, icddr, b, Dhaka, Bangladesh

**Keywords:** COVID-19, mortality, Bangladesh, elderly, sex

## Abstract

**Background:** Coronavirus disease 2019 (COVID-19) has spread globally, and the government of each affected country is publishing the number of deaths every day. This official figure is an underestimate as it excludes anybody who did not die in a hospital, who did not test positive, who had a false result, or those who recovered on their own without a test.

**Objective:** This study aimed to measure the community level excess mortality using health and demographic surveillance in a rural area of Bangladesh.

**Method:** The study was conducted in Matlab, in a rural area of Bangladesh, with a Health and Demographic Surveillance System (HDSS) covering a population of 239,030 individuals living in 54,823 households in 142 villages. We examined the mortality in January-April from 2015 to 2020 and compared the mortality in 2020 with the historical trend of 2015–2019. Between 2015 and 2020, we followed 276,868 people until migration or death, whichever occurred first. We analyzed mortality using crude mortality rate ratio (MRR) and adjusted MRR (aMRR) from a Cox proportional hazard model. Mortality was analyzed according to age, sex, and period.

**Results:** During follow-up, 3,197 people died. The mortality rate per 1,000 person-years increased from 10 in 2019 to 12 in 2020. Excess mortality was observed among the elderly population (aged 65 years and above). The elderly mortality rate per 1,000 person-years increased from 80 in 2019 to 110 in 2020, and the aMRR was 1.40 (95% CI: 1.19–1.64). Although an increasing tendency in mortality was observed between 2015 and 2019, it was statistically insignificant.

**Conclusions:** The study reported a 28% increase in excess deaths among the elderly population during the first months of the pandemic. This all-cause mortality estimation at the community level will urge policymakers, public health professionals, and researchers to further investigate the causes of death and the underlying reasons for excess deaths in the older age-group.

## Introduction

Coronavirus disease 2019 (COVID-19), caused by severe acute respiratory syndrome coronavirus 2 (SARS-CoV-2) (1), first emerged in Wuhan city of Hubei province in China on December 31, 2020, when Chinese health officials informed the WHO about a cluster of 41 patients with mysterious pneumonia, supposedly connected to Huanan Seafood Wholesale Market (2). The detection time of the first cases varied from country to country; some counties detected early in the pandemic and some later. For instance, Bangladesh announced the first cases on March 8, 2020 (Table 1), and 2 days later on March 11, 2020, WHO declared COVID-19 as a pandemic (2).

**Table 1:** Timeline of detection of first cases of COVID-19.

**Country**	**2019**	**2020**		
	**December**	**January**	**February**	**March**
China (3, 4)	31			
United Kingdom (5)		6		
Thailand (3, 4)		13		
Japan (4)		15		
Republic of Korea (South Korea) (4)		20		
United States (3)		20		
France (6)		24		
Germany (6)		27		
India (7)		27		
United Arab Emirates (UAE) (8)		29		
Spain (9)		31		
Iran (3, 10)			19	
Italy (3)			21	
Belarus, Lithuania, Netherlands, New Zealand, and Nigeria (11)			27	
Saudi Arabia (12)				2
South Africa (13)				5
Bangladesh (14)				8

The number of people migrating overseas from Bangladesh for employment annually is more than 400,000 (15). During the first months of 2020, more countries were placing lockdowns after the detection of the first cases of COVID-19 surfaced in each country. Bangladeshi migrant workers residing in these countries were compelled to return home to rural Bangladesh due to lack of income (16, 17). In addition, when Bangladesh announced its own lockdown on March 22, 2020 (18), after detecting the first three cases, an increasing number of people were leaving the capital city (19). Such movement of people opens the window for the spreading of the virus to more places in the country.

Official numbers of COVID-19 cases and deaths in Bangladesh are likely to be an underestimate of the real scenario because this mainly accounts for cases that have tested positive of coronavirus through laboratory confirmation (20) and deaths that are recorded in hospitals. What the numbers have missed could be cases of deaths and infections before testing started, and the first cases were confirmed when only six PCR laboratories were available throughout the country (21). After the tests were made available, false test results could have eliminated any real cases and unaccounted for people who did not step forward for tests fearing isolation and stigma (22) or those that recovered from taking treatment at home (23). In addition, it is also difficult to determine the cause of death in many instances to find out whether the person was COVID-19 positive if they died prior to testing (24).

Weak civil and vital registration statistics (CVRS) for the great majority of low- and middle-income countries (LMIC) (25) means that we have remarkably little insight into the magnitude of the excess mortality associated with the COVID-19 pandemic. This is in marked contrast to some high-income countries; for instance, during the first months of pandemic the United Kingdom published preliminary all-cause mortality data and COVID-19-related mortality data with a lag of only a few weeks unlike Bangladesh (26).

Although most countries are submitting daily data on the number of COVID-19 deaths to the WHO (27), without comparable all-cause mortality data, and all-cause mortality data for the equivalent time period over preceding years, it is difficult to estimate the excess mortality attributable to the pandemic. The excess mortality, of course, is not restricted to COVID-19 deaths alone; it also includes the non-COVID-19 deaths that arise from the loss of adequate care, as health systems become overstretched coping with COVID-19 cases.

Health and Demographic Surveillance System (HDSS) conducts surveillance of geographically prescribed populations for extended periods of time (28). They have, historically, provided estimates for many LMICs about the underlying birth, death, and fertility rates in the absence of an effective CVRS system (28). The Matlab HDSS is the longest running HDSS which has provided Bangladesh with some of its earliest data on rates of births and deaths (29). Utilizing these ongoing surveillance data, we are able to estimate the age and sex of mortality rates over the past 6 years and thereby to estimate the excess mortality in 2020 associated with the COVID-19 pandemic.

## Materials and Methods

### Settings and Population

The Matlab HDSS covers a population of 239,030 individuals living in 53,823 households in 142 villages in a rural district of Bangladesh that is situated 60 km south of Dhaka (30). All households are visited every 3 months to enquire about marriages, pregnancies, births, migrations, and deaths. Annually in the Matlab HDSS area, 5,298 babies are born, 1,687 people died, 2,671 people moved out of Bangladesh, and 1,371 people returned from abroad (30).

A web-based software application has been designed and developed for Matlab HDSS. Thirty-one tablets (smartphones) are connected to the mobile internet through the network of mobile operators. Traditionally, community health research workers (CHRWs) visit households every 3 months and record health and demographic events using these devices, and data are stored in the central database server. In the COVID-19 pandemic, to follow the precautionary guidelines in Bangladesh, CHRWs continued registering birth and death through mobile phones instead of household visits between March 25, 2020, and November 9, 2020. During data collection through mobile phones, CHRWs reached 85% of households in the first contact. Information of absent households (in the first contact) was updated in the next round.

### Epidemiological and Statistical Methods

**Figure 1:**
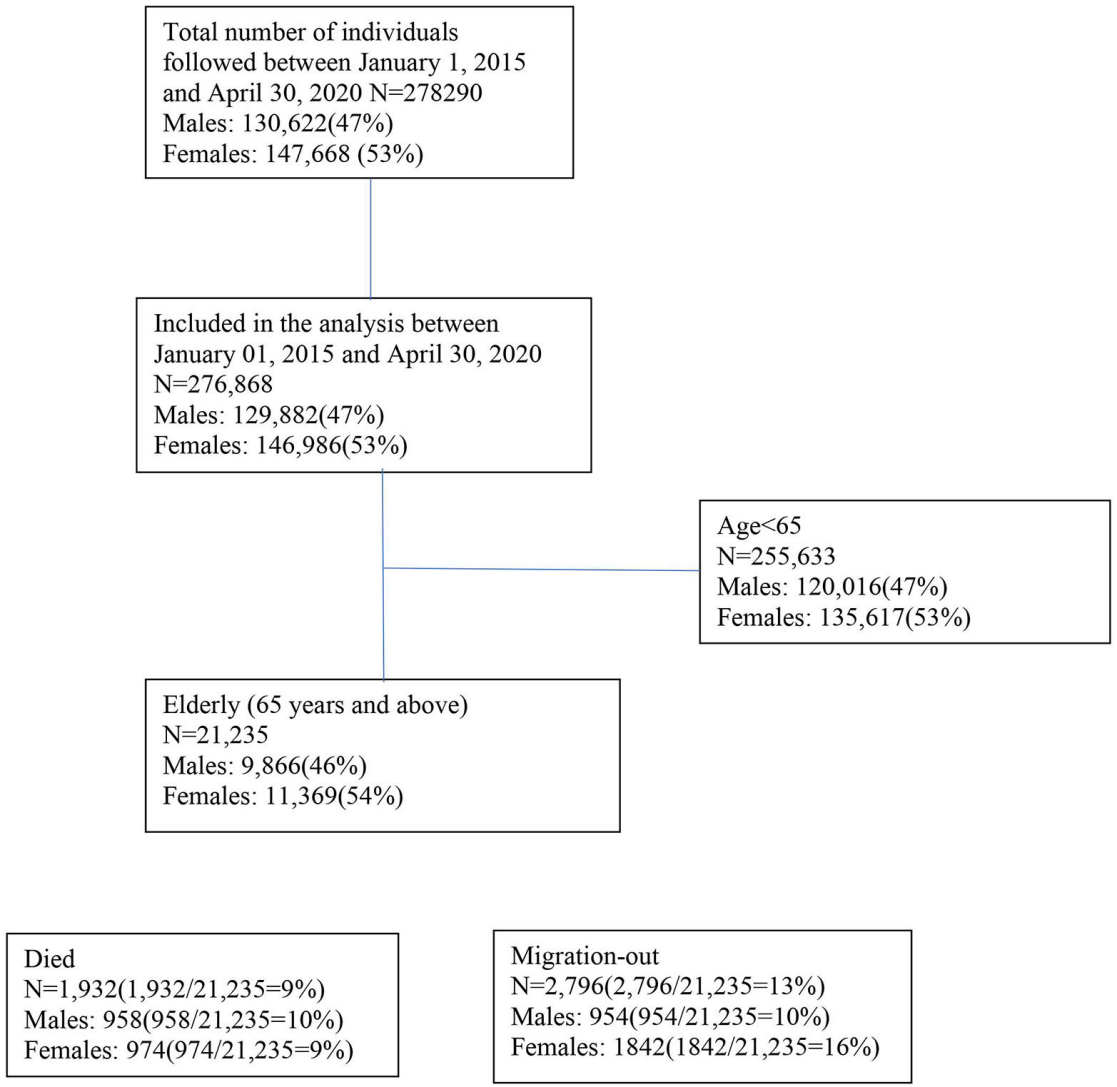
Flowchart of the study population.

We analyzed mortality data in the study area between January 2020 and April 2020 to take into account the transmission of coronavirus, not only related to the movement of population from the capital city to rural areas after declaring the first lockdown on March 22, 2020, in Bangladesh but also the spread of the virus exacerbated by the migrants returning from abroad during the first months of the pandemic (January 2020-April 2020) (24, 31). The mortality rate is compared with the mortality rates for the period of January 1 and April 30 in the years from 2015 to 2019. Between 2015 and 2020, we followed 278,290 people until migration or death, whichever occurred first (Figure 1). We calculated the mortality rate between January and April as seasonality in mortality was observed in the study area (30) (Figure 1). Deaths due to COVID-19 vary by age (32) and sex (33). We presented age-specific and sex-specific mortality and population size as the denominator. We used Cox proportional hazard models with age as the underlying timescale to estimate the aMRR. We included sex (33), period (30), and village (30) as potential confounders in the adjusted models.

## Results

### In-Migration

**Figure 2:**
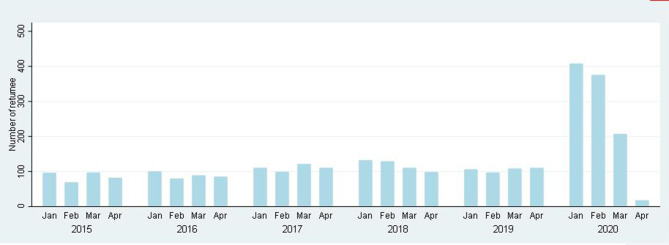
Number of people returned from abroad between 2015 and 2020.

In the Matlab HDSS area between January 2020 and April 2020, 1,008 people returned from abroad, which is 2.39 times (95% CI: 2.29–2.55) higher than in 2019. Moreover, in 2020, in-migration was very high, which is 3.87 times higher (95% CI: 3.10-4.14) in January and February compared to March and April (Figure 2).

### Mortality

**Figure 3:**
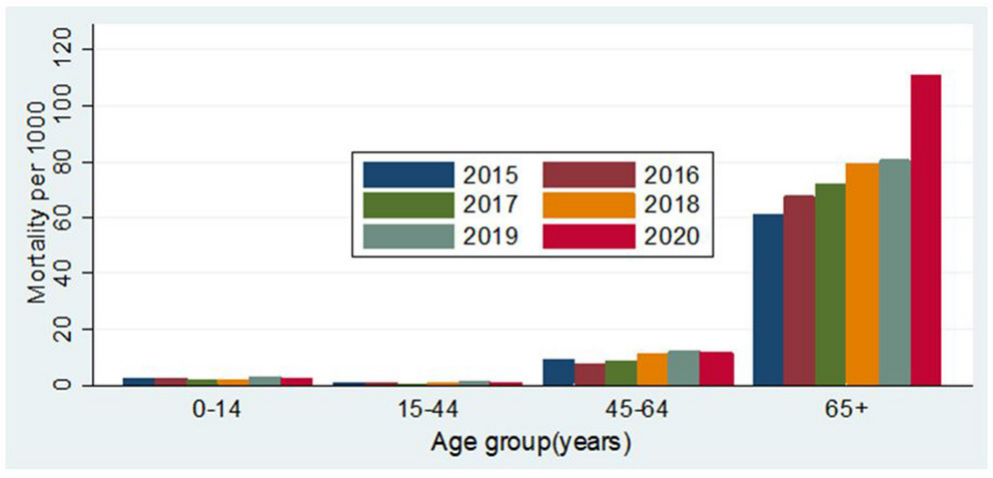
Mortality per 1,000 person-years according to age and year.

During the follow-up period from January to April from 2015 to 2020, a total of 3,197 people died. In 2020, the number of people migrating back to Matlab from abroad was 406 in January, 375 in February, 207 in March, and 17 in April. From 2015 to 2019, the crude mortality rates (CMRs) per 1,000 person-years were 7.37 (95% CI: 6.75–8.05), 7.63 (95% CI: 7.01–8.31), 7.81 (95% CI: 7.18–8.50), 8.98 (95% CI: 8.29–9.73), and 9.70 (95% CI: 8.99–10.48), respectively. In 2020, the CMR was 11.61 (95% CI: 10.51–12.82) (Table 2). Figure 3 shows the age by year mortality rates. Between 2015 and 2019, there is little evidence of substantial year-to-year variation in the mortality rate in any age-group. In 2020, the mortality rate in the 65+ age-group (older adults) is markedly higher than in the previous 5 years.

**Table 2:** Mortality rate per 1,000 person-years according to age, sex, and calendar year.

**Age group (in years)**	**Male**						^*^P-value	**Female**						^*^P-value
	**Mortality rate (deaths/pyrs)**							**Mortality rate (deaths/pyrs)**						
	**2015**	**2016**	**2017**	**2018**	**2019**	**2020**		**2015**	**2016**	**2017**	**2018**	**2019**	**2020**	
0–14	3(36/11,102)	3(38/11179)	3(28/10,988)	3(29/10,787)	4(44/10,472)	4 (19/5,406)	0.440	2(22/10,999)	2(20/10,992)	2(20/10,992)	2(24/10,808)	3(27/10,527)	3 (14/5,393)	0.397
15–44	1(16/11,618)	1(16/11795)	1(12/11,521)	2(20/10,965)	1(15/10,347)	1 (5/5,258)	0.997	1(13/15,891)	1(13/16,399)	1(13/16,399)	1(14/16,314)	2(25/15,826)	1 (8/8,120)	0.148
45–64	12(78/6,588)	9 (62/6652)	12(79/6,605)	15(98/6,454)	16(102/6,367)	16(51/3,285)	0.001	7 (51/7,207)	6 (45/7,154)	6 (45/7,154)	7 (49/6,960)	9 (59/6,713)	8 (28/3,500)	0.138
65+	67(144/2,153)	68(151/2205)	86(183/2,133)	88(185/2,102)	84(169/2,015)	117(126/1,081)	0.000	56(142/2,539)	60(154/2,560)	60(154/2,560)	72(182/2,524)	77(186/2,405)	105(136/1,289)	0.000
Total	9(274/31,461)	8(267/31830)	10(302/31,247)	11(332/30,308)	11(330/29,201)	13(201/15,030)	0.000	6(228/36,635)	7(261/37,349)	6(232/37,105)	7(269/36,606)	8(297/35,471)	10(186/18,303)	0.000

* *Test for the trend in mortality from 2015 to 2020*.

The mortality rate in older adults per 1,000 person-years increased from 80 in 2019 to 110 in 2020, and the aMRR was 1.39 (95% CI: 1.17–1.66) among older adults. The aMRR was 1.38 (95% CI: 1.09–1.75) for men and 1.43 (95% CI: 1.16–1.77) for women. No significant difference was observed between men and women in increasing mortality from 2019 to 2020 (*p* = 0.910). It should be noted that the apparent increasing tendency in mortality observed in the 65+ age-group between 2015 and 2019 was not statistically significant (Table 1).

## Discussion

### Main Findings

Compared to the five previous years, there is a clear, statistically significant difference in the mortality rate in the 65+ age-group during the COVID-19 period in Matlab. This result is strong indication of a pandemic effect. No such difference was observed in any of the younger age-groups. The age by mortality effect is consistent with the global data on excess mortality that shows the highest mortality risk in those who are aged 65 years or older (32, 34, 35).

### Interpretation

The COVID-19 pandemic has had a significant negative impact on mortality rates in older people living in rural Bangladesh. It is not possible at this stage to determine the cause of death—although a verbal autopsy process, to be conducted in a few months, may give some insight into this. However, given the age effect in the excess mortality, the results strongly indicate either a direct or an indirect COVID-19 effect. The indirect effect may be attributable to a decreased likelihood of seeking life-saving care or a decreased capacity of the health system to manage non-COVID-19-related healthcare needs (36). For instance, the prevalence of hypertension is 53% among older age-groups (aged 65 years and above) in Bangladesh (37) and these hypertensive patients may not have been able to avail regular checkups or acquire medicines during the pandemic. Moreover, about 50% of deaths occur in the study area per year (30) due to conditions of chronic disease like heart disease and stroke. The pandemic situation may be responsible for deaths among such patients by preventing them from traveling the distance to a hospital that could provide them the immediate intensive care services that they required.

### Strength and Weakness

We analyzed mortality from January to April, 2015–2020, which reduced any seasonality bias (30). Mortality rates were calculated using person-time techniques (38) that remain the basic epidemiological approach to estimating mortality, yet one of them is frequently missed in the calculation of mortality (39).

The data were only collected from one rural area, which may not reflect the situation in all rural Bangladesh. Indeed, Matlab appears to have better health outcomes than other rural areas of Bangladesh, and this may indicate that excess mortality rates would be worse elsewhere. The relatively short period of observation was to ensure the timely reporting of data.

We cited a number of news media-released reports as there was no detailed timeline information on these events during the first months of the pandemic.

## Conclusions

Globally, the COVID-19 pandemic impacted the mortality of the overall population. COVID-19 pandemic attributed 30 deaths per 1,000 among the older age-group in the study area. We did not determine the deaths related to COVID-19 and non-COVID-19 causes. A further cause of death analysis will provide an estimate of excess deaths associated with COVID-19 and non-COVID-19 causes. It is important to examine whether the excess deaths as a result of access to healthcare and how the national COVID-19 policy operates on the decisions and actions of the people. Bangladesh needs to strengthen the CVRS system and national health statistics to monitor timely morbidity and mortality, especially in an epidemic or pandemic situation. Bangladesh should strive to strengthen its health systems by providing additional resources to make healthcare services more accessible to its residents irrespective of geographical locations.

## Data Availability Statement

The raw data supporting the conclusions of this article will be made available by the authors, without undue reservation.

## Ethics Statement

The studies involving human participants were reviewed and approved by Ethical Review Committee of icddr,b. The patients/participants provided their written informed consent to participate in this study.

## Author Contributions

SH and DR conceived and designed the study and are the guarantors of the study. SA prepared the data file. SH analyzed the data and wrote the first draft of the manuscript. SS contributed to the literature review. All authors contributed to the final version of the manuscript. All authors had full access to all of the data (including statistical reports and tables) in the study and can take responsibility for the integrity of the data and the accuracy of the data analysis.

## Conflict of Interest

The authors declare that the research was conducted in the absence of any commercial or financial relationships that could be construed as a potential conflict of interest.

## Publisher's Note

All claims expressed in this article are solely those of the authors and do not necessarily represent those of their affiliated organizations, or those of the publisher, the editors and the reviewers. Any product that may be evaluated in this article, or claim that may be made by its manufacturer, is not guaranteed or endorsed by the publisher.
